# Knock down of Fas-Associated Protein with Death Domain (FADD) Sensitizes Osteosarcoma to TNFα-induced Cell Death

**DOI:** 10.7150/jca.38721

**Published:** 2020-01-14

**Authors:** Mario G. Hollomon, LaNisha Patterson, Janice Santiago-O'Farrill, Eugenie S. Kleinerman, Nancy Gordon

**Affiliations:** 1 Department of Biology, Texas Southern University, Houston, TX 77004; 2 Department of Neuroscience, Cell Biology and Anatomy, The University of Texas Medical Branch, Galveston, TX 77555; 3 Division of Experimental Therapeutics, The University of Texas MD Anderson Cancer Center, Houston, TX 77054; 4 Division of Pediatrics, The University of Texas MD Anderson Cancer Center, Houston, TX 77054

**Keywords:** FADD, osteosarcoma, TNFα, NFκB, XIAP

## Abstract

Fas-associated protein with death domain (FADD) was first identified for its role in linking death receptors to the apoptotic signaling pathway with subsequent cell death. Later studies reported non-apoptotic functions for FADD in normal cells and cancer cells. Non-apoptotic functions for FADD in osteosarcoma (OS) have not been reported. In this study, FADD protein expression was knocked down in human CCHOSD, LM7, and SaOS2 OS cell lines followed by assessment of sensitivity to TNFα- or TRAIL-induced cell death. Knock down of FADD significantly increased TNFα-induced cell death in LM7 and SaOS2 cell lines. The mode of TNFα-induced cell death was apoptosis and not necroptosis. Inhibition of nuclear factor kappa B (NFκB) in wildtype cells increased TNFα-induced cell death to similar levels observed in FADD knockdown cells, suggesting a role for FADD in NFκB pro-survival cell signaling. In addition, knock down of FADD increased SMAC mimetic-mediated TNFα-induced cell death in all cell lines studied. The results of this study indicate that FADD has a pro-survival function in OS following TNFα treatment that involves NFκB signaling. The results also indicate that the pro-survival function of FADD is associated with XIAP activity.

## Introduction

Osteosarcoma (OS) is the most common type of bone cancer found in children and teens 1. The long-term survival rate for localized OS is approximately 65 percent 2 while the long-term survival rate for OS that has spread to other parts of the body is less than 30 percent 3. These long-term survival rates underscore the need for better therapeutic options for OS. Development of improved therapies for OS requires a better understanding of the pro-survival and pro-death signaling pathways within OS.

Fas-associated protein with death domain (FADD) was discovered as an adaptor protein that interacts with the intracellular death domain (DD) of the Fas receptor (Fas) following ligation of Fas with Fas ligand (FasL) with subsequent apoptosis 4. Studies later identified additional FADD interacting proteins. For example, upon TNF-related apoptosis-inducing ligand (TRAIL) receptor ligation, FADD binds the intracellular DD of the TRAIL receptor 5. Additionally, upon TNF receptor-1 (TNFR1) ligation with TNFα, FADD binds TNFR-associated death domain (TRADD) protein 6. Following interaction of FADD with Fas, TRAIL receptor or TRADD, FADD recruits procaspase-8 via its death-effector-domain (DED) with subsequent activation of the extrinsic apoptotic pathway.

Subsequent studies on FADD revealed non-apoptotic functions for FADD. For example, FADD has been reported to promote cell proliferation and cell cycle regulation in T cells 7, 8. These reports were among the first to show that FADD has non-apoptotic functions. Later studies reported that FADD has non-apoptotic functions beyond T cells. For example, FADD has been reported to protect pancreatic cancer cells from anticancer drug-induced cell death 9.

The tumor necrosis factor super family (TNFSF) of ligands is a diverse group of cytokines that induce a variety of responses from inflammation to apoptosis. TNFSF ligands include TNFα, TRAIL and FasL. These ligands are also referred to as death ligands and their cognate receptors are referred to as death receptors. TNFα is the most pleiotropic member of the TNFSF ligand family. TNFα is a pro-inflammatory cytokine secreted by multiple immune cells. TNFα promotes cancer cell growth, angiogenesis, metastasis and apoptosis 10.

The NFκB family of proteins consist of five members: p105/50, p110/52, RelB, RelA(p65) and c-Rel. Active NFκB transcription factor exist as a homodimer or heterodimer, with p50/p65 representing the prominent dimer. Upon activation of the NFκB pathway, the NFκB inhibitor, inhibitor of NFκB (IκB), is phosphorylated by IκB kinase (IKK) causing the release of NFκB and translocation to the nucleus where NFκB serves as a transcription factor. NFκB is primarily associated with expression of pro-survival genes such as Bcl-2 11, c-flip 12, and XIAP 13.

X-linked inhibitor of apoptosis (XIAP) is a target gene of NFκB that promotes cell survival following certain cell death-inducing stimuli. XIAP inhibits apoptosis by binding to activated caspases-3, -7 and -9 14. Therefore, XIAP is a negative regulator of apoptosis. Endogenous inhibition of XIAP is achieved by second mitochondrial-derived activator of caspases (SMAC). SMAC binds XIAP, thus preventing XIAP-mediated inhibition of apoptosis. The ability of SMAC to inhibit XIAP was the basis for the investigation of SMAC mimetics as a therapeutic option for cancer 15.

The role of FADD in death ligand signaling and death ligand-induced cell death in OS has not been well characterized. Here, we report that knock down of FADD sensitizes OS to TNFα-induced cell death. We also report that inhibition of NFκB increases TNFα-induced cell death. In addition, we report that inhibition of XIAP increases TNFα-induced cell death in FADD knockdown cells. The results of this study indicate that FADD has a pro-survival role in OS following TNFα treatment that involves NFκB activation and XIAP activity.

## Materials and Methods

### Antibodies and Reagents

TNFα and TRAIL cytokines were purchased from Peprotech (Rocky Hill, NJ). Antibodies against cleaved caspase-3, phospho-inhibitor of NFκB (pIκB), p50, p65, Lamin B and FADD were purchased from Cell Signaling Technology, Inc. (Danvers, MA). Pan-caspase inhibitor (Z-VAD-FMK) was purchased from Enzo Life Sciences (New York, NY). Ripa lysis buffer was purchased from Santa Cruz Biotechnology, Inc. (Dallas, TX). Necrostatin-1, PS-1145 and actin antibody were purchased from Sigma Aldrich (St. Louis, MO). SM-164 was purchased from ApexBIO (Houston, TX). Fetal bovine serum (FBS) was purchased from Atlanta Biologicals (Lawrenceville, GA). Dulbecco's modified eagle medium (DMEM) cell culture medium and cell culture supplements were purchased from Invitrogen (Carlsbad, CA).

### Cell Lines and Cell Culture

CCHOSD, LM7 and SaOS2 are human OS cell lines. CCHOSD is a human metastatic osteosarcoma cell line. LM7 is the human high metastatic 16 subline of the low metastatic potential SaOS2 cell line 17. Prior to experimentation, cells were fingerprinted by short tandem repeat analysis of DNA by the Characterized Cell Line Core Facility at The U.T. MD Anderson Cancer Center. Cells were tested for mycoplasma contamination using Lonza mycoplamsa detection kit (Saint Beauzire, France). Cells were cultured in DMEM containing 10% FBS and supplemented with antibiotic, non-essential amino acid solution, MEM vitamin mixture, and cultured in an incubator maintained at 5% CO_2_ and 37^o^C.

### Generation of FADD knockdown cells

Lentiviral shRNA (GE Dharmacon, Lafayette, CO) targeted to FADD RNA was used to knock down FADD protein expression. Lentivirus was generated by transfecting 293T cells with 7ug/ml transfer plasmid (shRNA plasmid), 5ug/ml psPAX2 (packaging plasmid) and 4ug/ml pMD2.G (envelope plasmid). Forty-eight hours after 293T cell transfection, supernatant containing lentivirus was collected and immediately used for infection or stored at -80^o^C. For infection, 2 ml of supernatant containing lentivirus were added to each well of a 6-well plate containing 2x10^5^ cells. Cells were incubated with lentivirus for 8 h. Efficiency of FADD protein knock down was determined 72 h following infection by assessing FADD protein levels via western blot analysis. Two FADD knockdown cell lines were generated using two different lentiviral shRNA sequences [TRCN0000040268, TRCN0000040269]. Cells infected with empty shRNA vector (no shRNA-specific insert) are hereafter referred to as wildtype (wt). Cells infected with shRNA targeted against FADD RNA are hereafter referred to as FADD knockdown (fkd).

### Cell Viability

To determine cell viability, 1x10^5^ cells/well were seeded in 12-well plates and treated with drug 24 h later as indicated in figure legends. Following drug treatment, floating cells and attached cells were collected followed by cell viability determination. Cell viability was determined by trypan blue exclusion assay using an automated cell counter (Vi-Cell, Beckman Coulter, Miami, FL). Cells restricting trypan blue entry were considered viable.

### Western blot

Following drug treatment, floating cells and attached cells were collected and centrifuged at 1000 rpm for 5 min at 4^o^C. The resultant pellet was lysed with RIPA lysis buffer containing protease and phosphatase inhibitor cocktail and centrifuged at 12,000 rpm for 10 min at 4^o^C. Supernatants were then collected and total protein was determined by BioRad reagent (BioRad Laboratories, Hercules, CA). Where applicable, cytoplasmic and nuclear fractions were separated by use of a nuclear extraction kit according to manufacturer's instructions (Cayman Chemical, Ann Arbor, MI). Fifty micrograms of protein were resolved in SDS-polyacrylamide gels (SDS-PAGE) and transferred onto nitrocellulose membranes (BioRad Laboratories, Hercules, CA). Membranes were next blocked with 5% nonfat milk followed by incubation with antibodies against FADD, cleaved caspase-3, pIκB, p50, p65, Lamin B or beta-actin. Membranes were next washed and incubated with appropriate secondary antibody conjugated to HRP (GE Healthcare Life Sciences, Piscataway, NJ). Following secondary antibody incubation, membranes were washed and signal detected with ECL detection reagent (GE Healthcare Life Sciences, Piscataway, NJ). Lamin B served as a nuclear marker. Beta-actin served as a protein loading control.

### TNFα receptor expression

Cells were grown to approximately 70% confluency followed by removal with trypsin and washed twice with PBS. Cells were next treated with PE-conjugated TNFR1 antibody or PE-conjugated IgG control antibody. Following antibody treatment, cells were washed twice and surface TNFR1 expression analyzed by flow cytometry.

### Statistical Analysis

Results are presented as means ± standard error mean (SEM). Experimental data were analyzed using 2-tailed Student *t* test. P-values <0.05 were considered statistically significant and is indicated by an asterisk.

## Results

### Knock down of FADD protein increases sensitivity to TNFα

**Figure 1:**
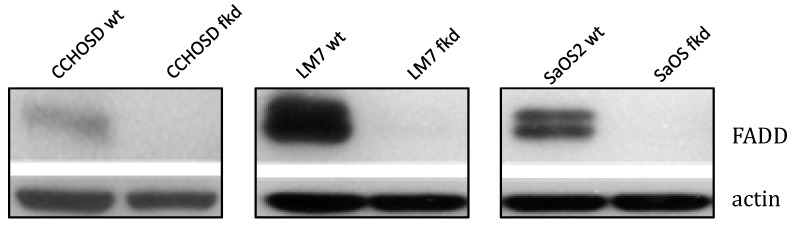
** Lentiviral shRNA directed against FADD effectively knocks down FADD protein expression.** Cells were infected with shRNA lentivirus targeted against FADD RNA. Following infection, FADD protein levels were determined by western blot analysis. Beta-actin served as a protein loading control.

**Figure 2:**
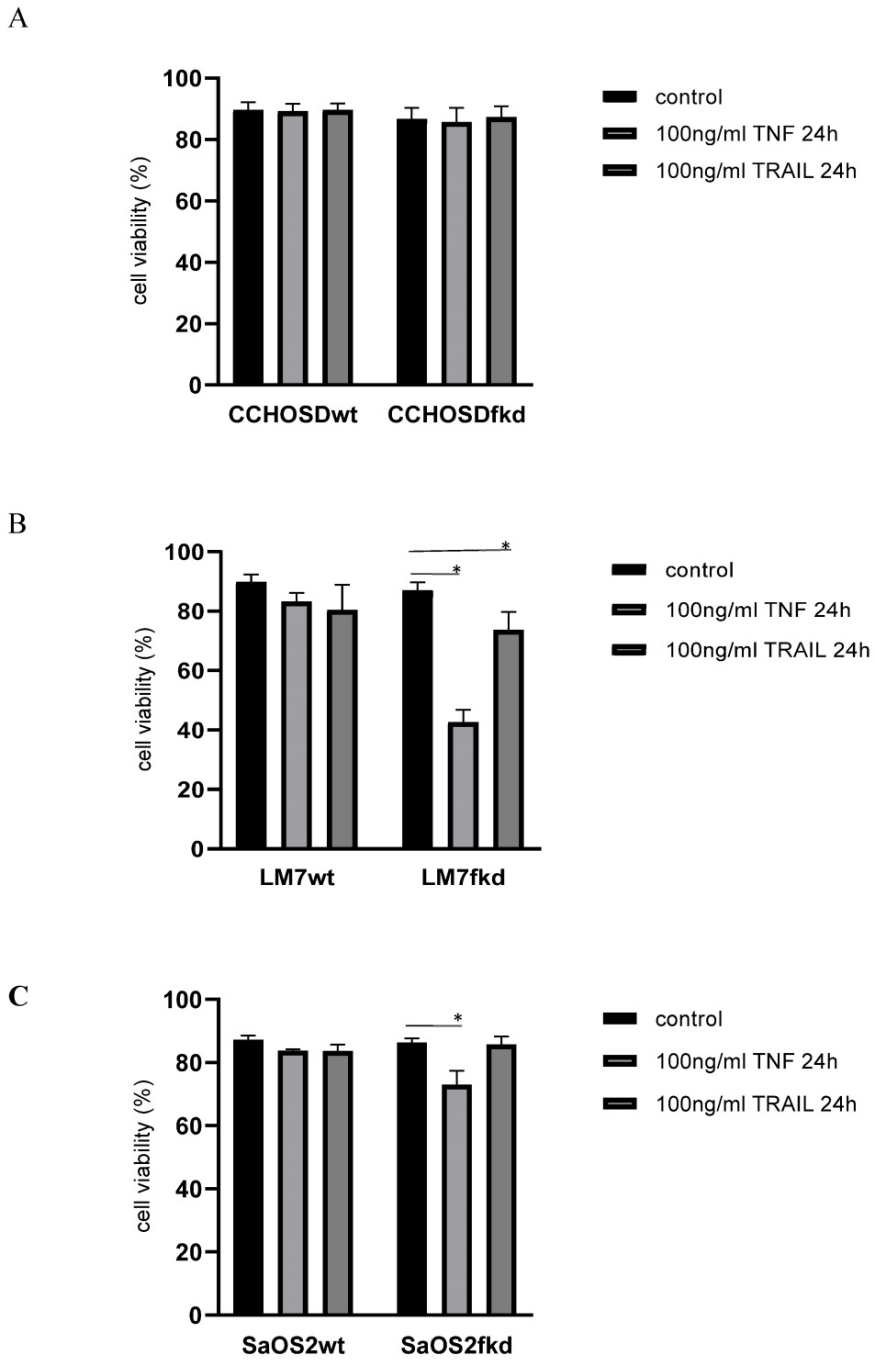
** Knock down of FADD increases TNFα-induced cell death.** Cells were treated with 100ng/ml TNFα or 100ng/ml TRAIL for 24 h. Following death ligand treatment, cell viability was determined by trypan blue exclusion assay. **A**, CCHOSD. **B**, LM7. **C**, SaOS2. Data represents the results of at least three independent experiments, ± SEM. *, p<0.05 was considered significant.

**Figure 3:**
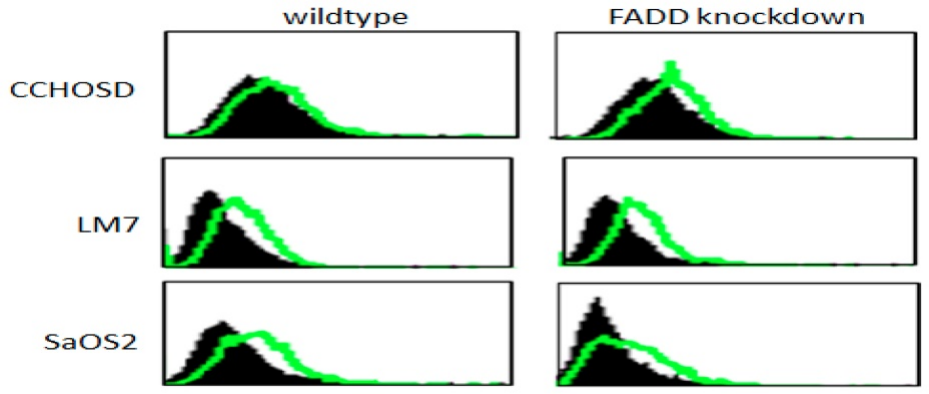
** TNFα receptor surface expression.** Untreated wildtype and FADD knockdown cells were incubated with PE-labeled TNFR1 antibody. TNFα receptor surface expression was analyzed by flow cytometry. Filled histogram plot: IgG control. Unfilled histogram plot: TNFR1 expression.

Following confirmation of FADD knockdown (Figure 1), cells were treated with TNFα or TRAIL. Cell death in TNFα-treated wildtype CCHOSD (CCHOSDwt) or FADD knockdown CCHOSD (CCHOSDfkd) cells was unchanged (Figure 2A). TNFα treatment induced significant cell death in FADD knockdown LM7 (LM7fkd) and FADD knockdown SaOS2 (SaOS2fkd) cells (Figure 2B-C). TRAIL treatment induced significant cell death in LM7fkd cells (Figure 2B). To determine if FADD knockdown affected TNFα receptor (TNFR1) expression, TNFR1 expression was assessed. Knock down of FADD did not alter surface expression of TNFR1 (Figure 3).

### Caspase inhibition, but not necroptosis inhibition, reverses TNFα-induced cell death

**Figure 4:**
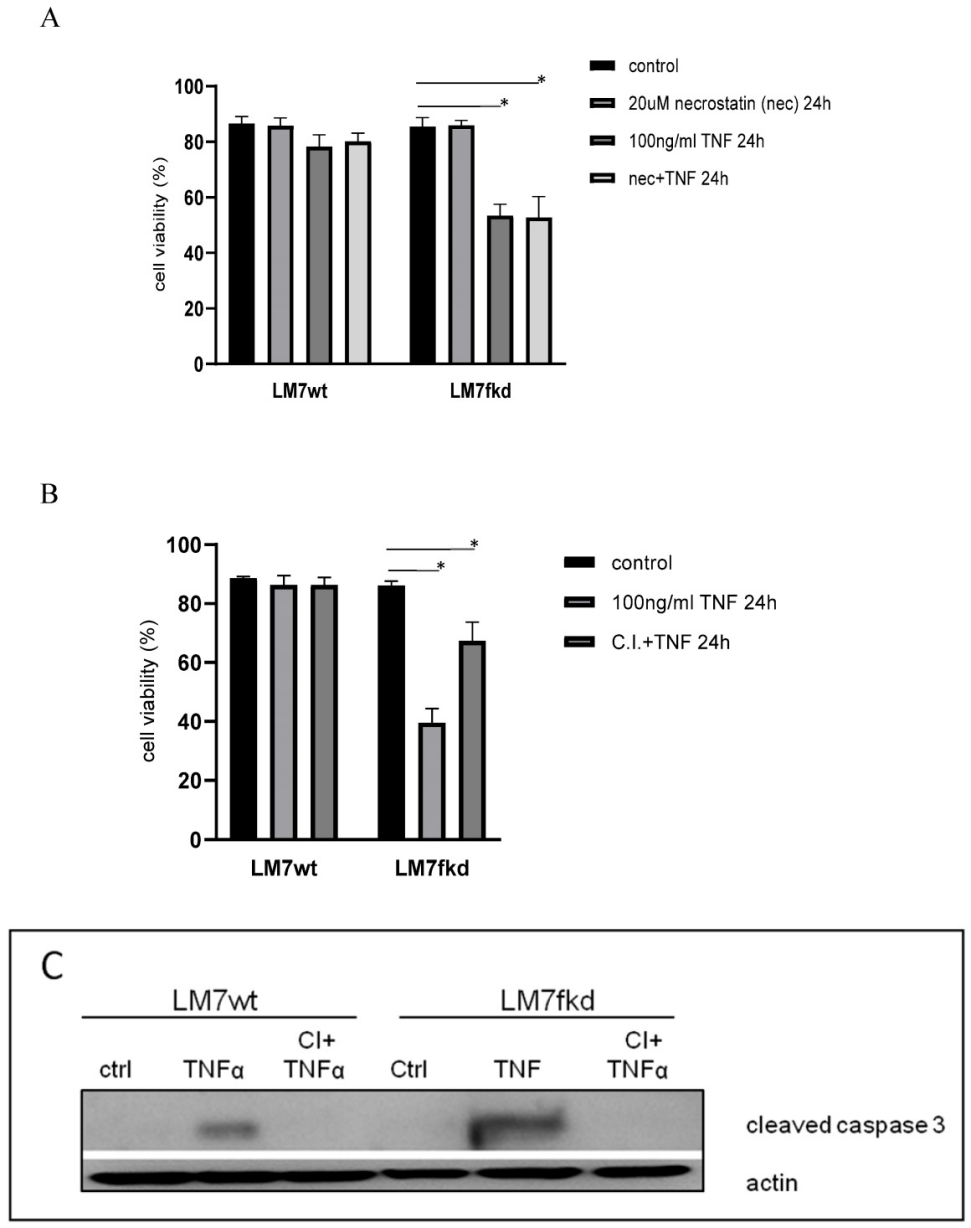
** Inhibition of caspases, but not necroptosis, reverses TNFα-induced cell death. A**, Inhibition of necroptosis does not reverse TNFα-induced cell death. Cells were pretreated with 20uM necrostatin-1 for 2 h followed by 100ng/ml TNFα treatment for 24 h. **B**, Inhibition of caspases reverses TNFα-induced cell death. Cells were pretreated with 30uM pan-caspase inhibitor for 2 h followed by 100ng/ml TNFα treatment for 24 h. Cell viability was determined by trypan blue exclusion assay. Data represents the results of at least three independent experiments, ± SEM. *, p< 0.05 was considered significant. **C**, TNFα treatment causes caspase-3 activation in LM7wt and LM7fkd cells. Pan-caspase inhibitor pretreatment blocks TNFα-induced caspase-3 activation. Immunoblot is representative of immunoblots from three independent experiments.

The mode of cell death responsible for TNFα-induced cell death in LM7fkd cells where TNFα induced the most significant cell death was investigated. TNFα has been reported to cause necroptosis 18. Therefore, necroptosis was initially investigated as the mode of TNFα-induced cell death. LM7wt and LM7fkd cells were pretreated with the necroptosis inhibitor, necrostatin-1, followed by TNFα treatment. Pretreatment with necrostatin-1 did not rescue LM7fkd cells from TNFα-induced cell death (Figure 4A), suggesting that necroptosis was not the mode of cell death for TNFα-induced cell death in LM7fkd cells. However, pretreatment of LM7fkd cells with a pan-caspase inhibitor (Z-VAD-FMK) followed by TNFα treatment reversed TNFα-induced cell death, suggesting apoptotic cell death (Figure 4B). Pan-caspase inhibitor effectively blocked TNFα-induced caspase-3 activation. Caspase-3 activation was observed in both LM7wt and LM7fkd cells following TNFα treatment (4C).

### Inhibition of NFκB activation increases TNFα-induced cell death

**Figure 5:**
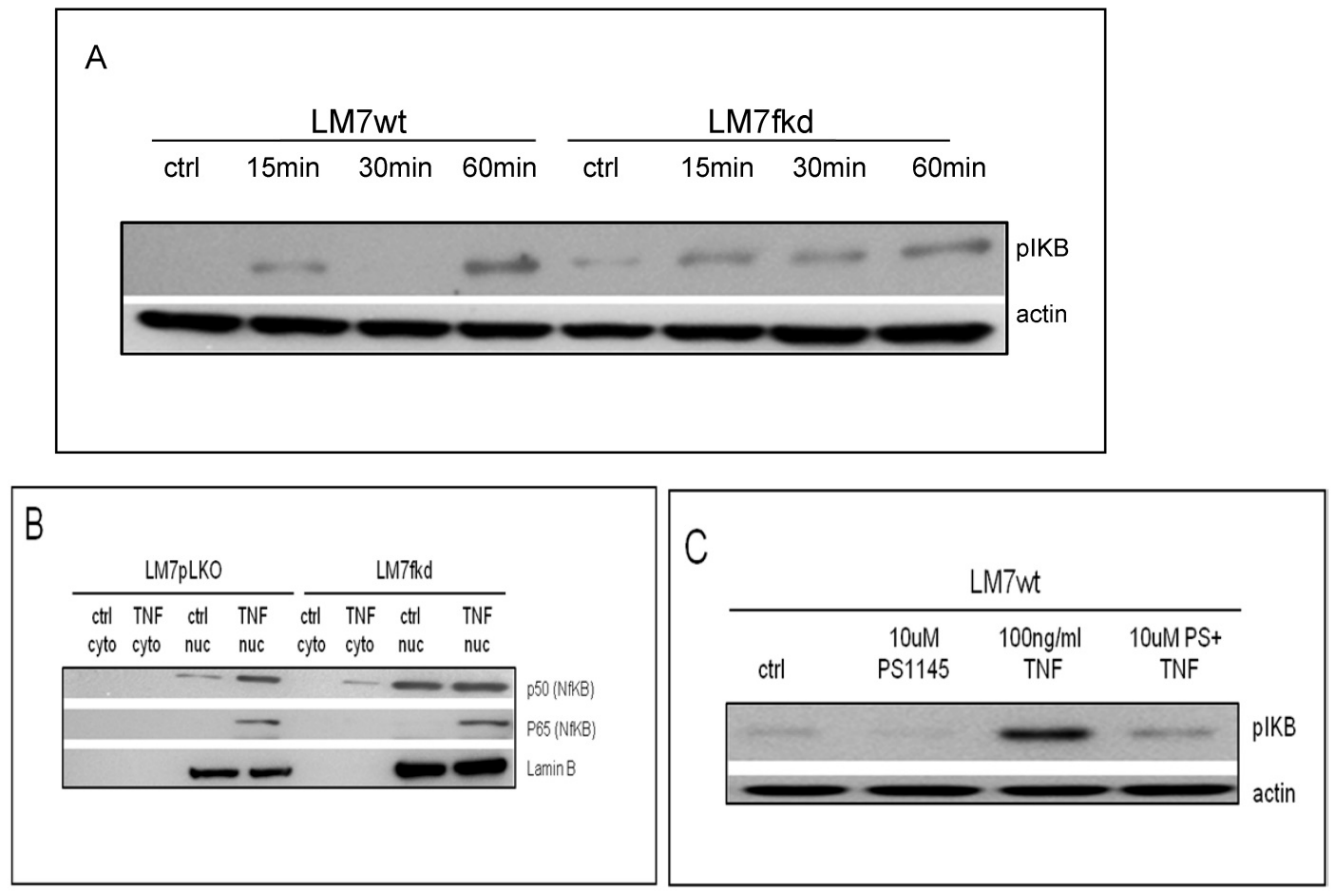
** Functional status of NFκB signaling. A**, IkB phosphorylation following TNFα treatment. Cells were treated with 100ng/ml TNFα for times indicated in figure. Following TNFα treatment, cells were collected, lysed and total protein probed for pIkB. Beta-actin served as a loading control. Immunoblot is representative of immunoblots from three independent experiments. **B**, Translocation of p50 and p65 to the nucleus following TNFα treatment. Cells were treated with 100ng/ml TNFα for 60 min. Following TNFα treatment, cells were collected and nuclear and cytoplasmic fractions separated. Lamin B served as the nuclear marker. Immunoblot is representative of immunoblots from two independent experiments. **C,** PS-1145 blocks phosphorylation of IkB. Cells were pretreated with 20uM PS-1145 for 2 h followed by treatment with 100ng/ml TNFα for 60 min. Following TNFα treatment, cells were collected, lysed and total protein probed for pIkB. Beta-actin served as a protein loading control. Immunoblot is representative of immunoblots from three independent experiments.

**Figure 6:**
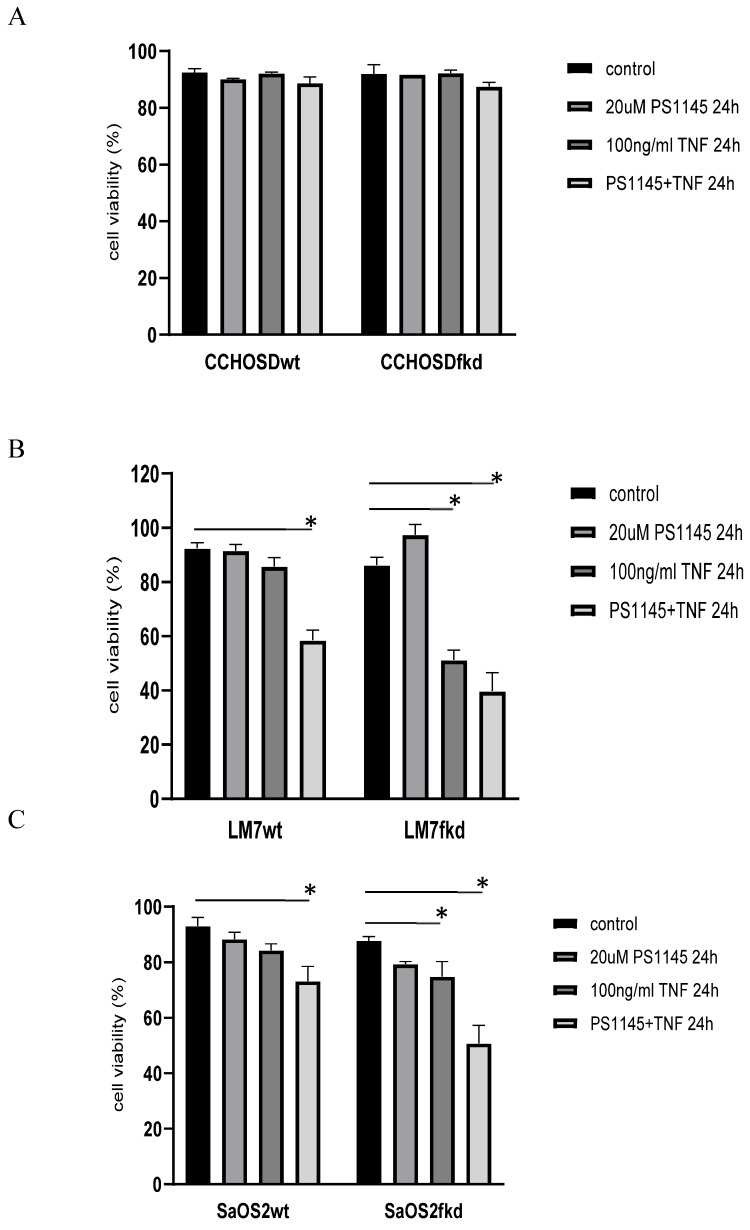
** Inhibition of IκB kinase (IKK) increases TNFα-induced cell death.** Cells were pretreated with the IKK inhibitor, PS-1145, for 2 h followed by treatment with 100ng/ml TNFα for 24 h. Cell viability was determined by trypan blue exclusion assay. **A,** CCHOSD, **B,** LM7, **C,** SaOS2. Data represents the results of at least three independent experiments, ± SEM. *, p< 0.05 was considered significant.

TNFα treatment induced phosphorylation of IkB in both LM7wt and LM7fkd cells (Figure 5A). NFκB activation and functional status of the NFκB signaling pathway was confirmed by the translocation of p50 and p65 to the nucleus following TNFα treatment (Figure 5B). PS-1145 inhibits IKK, thus preventing NFκB activation. Pretreatment with PS-1145 reversed TNFα-induced IkB phosphorylation (Figure 5C), suggesting inhibition of NFκB. Therefore, to investigate the effect of NFκB inhibition on TNFα treatment, wildtype and FADD knockdown OS cells were pretreated with PS-1145 followed by TNFα treatment. Pretreatment with PS-1145 significantly increased TNFα-induced cell death in LM7wt cells to similar levels observed in LM7fkd cells treated with TNFα alone (Figure 6B), suggesting that the TNFα-induced cell death observed in LM7fkd cells was associated with NFκB activation. Inhibition of NFκB also increased TNFα-induced cell death in SaOS2fkd cells to similar levels observed in LM7fkd cells treated with TNFα alone (Figure 6C).

### Inhibition of XIAP increases TNFα-induced cell death in FADD knockdown cells

**Figure 7:**
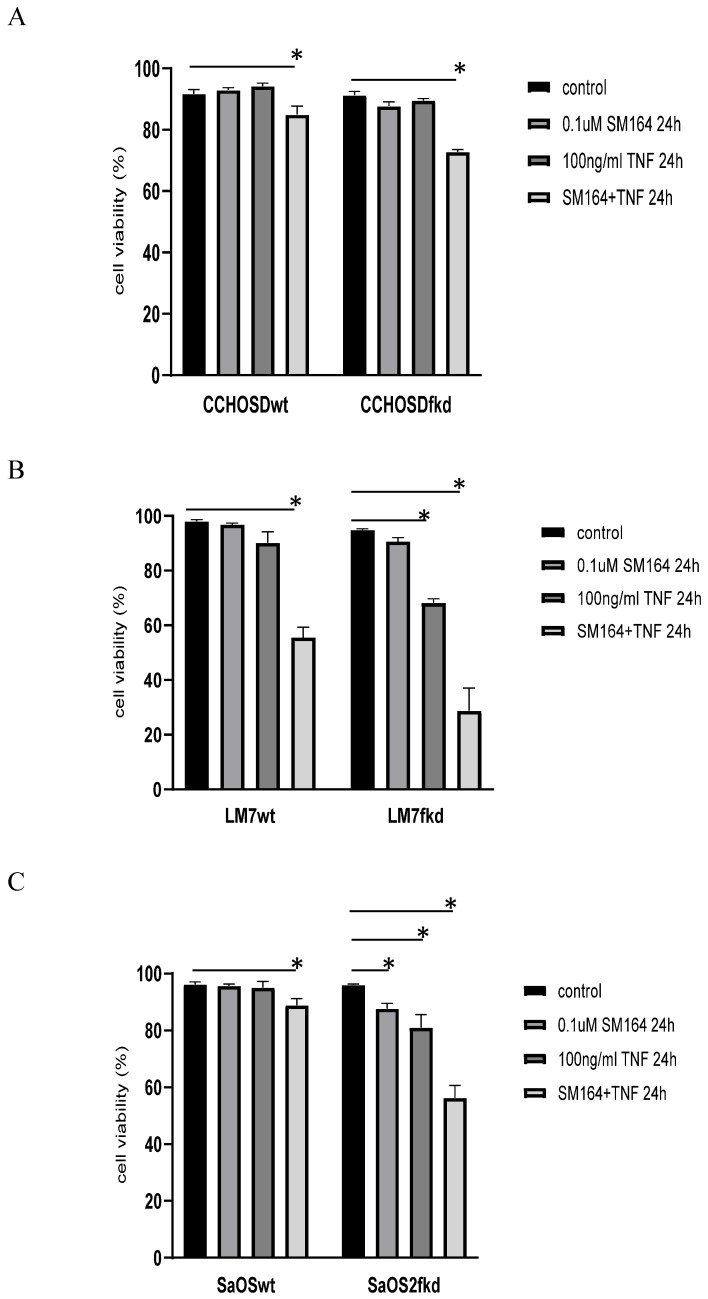
** Knock down of FADD increases TNFα-induced cell death in XIAP-inhibited cells.** Cells were pretreated with SM-164 for 2 h followed by treatment with 100ng/ml TNFα. Cell viability was determined by trypan blue exclusion assay. **A,** CCHOSD, **B,** LM7, **C,** SaOS2. Data represents the results of at least three independent experiments, ± SEM. *, p< 0.05 was considered significant.

To investigate the effect of XIAP inhibition on TNFα treatment in FADD knockdown cells, wildtype and FADD knockdown OS cells were pretreated with the XIAP inhibitor, SM-164, followed by TNFα treatment. FADD knockdown increased SM-164-mediated TNFα-induced cell death in all OS cell lines investigated (Figure 7A-C).

## Discussion

FADD was first identified as an adaptor protein that links Fas to caspase-8 with subsequent activation of apoptosis and subsequent cell death. Subsequent studies reported non-apoptotic functions for FADD. The early non-apoptotic functions for FADD were reported in T cells 7, 8. Later studies reported non-apoptotic or pro-survival functions for FADD beyond T cells, to include cancer cells 9. While FADD has recently been reported to have a protective role against anticancer drug-induced cell death in pancreatic cancer cells 9, there are no reports on the effect of FADD knockdown on cell death in OS induced by death ligands such as TNFα or TRAIL. This study set out to investigate the effect of TNFα on cell death in OS cells with FADD knockdown. The death ligand TRAIL was also investigated as a comparison death ligand.

In the present study, knock down of FADD in LM7 or SaOS2 cells caused significant cell death following TNFα treatment. The initially reported role of FADD in death receptor-initiated apoptosis suggests that knock down of FADD should inhibit death ligand-induced apoptosis. Therefore, considering the report that TNFα triggers necroptosis in cells with an inhibited apoptotic pathway 19, 20, necroptosis was investigated as the mode of TNFα-induced cell death in LM7fkd cells following TNFα treatment. Necroptosis is a form of cell death referred to as programmed necrosis and TNFα is the principal inducer of necroptosis 21. LM7wt and LM7fkd cells were pretreated with the necroptosis inhibitor, necrostatin-1, followed by TNFα treatment. Necrostatin-1 pretreatment did not reverse TNFα-induced cell death (Figure 4A), suggesting another mode of cell death. Apoptosis was next investigated as the mode of cell death by pretreating cells with a pan-caspase inhibitor followed by TNFα treatment. Pan-caspase inhibition reversed the TNFα-induced cell death in LM7fkd cells, indicating caspase-mediated cell death, or apoptosis, as the mode of TNFα-induced cell death (Figure 4B). To further confirm apoptosis, LM7wt and LM7fkd cells were probed for activated caspase-3 following TNFα treatment. Considering the insignificant cell death observed in LM7wt cells following TNFα treatment, we expected that activation of caspase-3 would be restricted to TNFα-treated LM7fkd cells. As expected, activated caspase-3 was detected in LM7fkd cells. Surprisingly, an appreciable level of activated caspase-3 was observed in LM7wt cells (Figure 4C).

Considering the observation in the present study that FADD knockdown increases TNFα-induced cell death and the report that TNFα activates the NFκB pathway 22, the effect of NFκB inhibition in FADD knockdown OS cells was investigated. NFκB was inhibited in wildtype and FADD knockdown OS cells followed by TNFα treatment. Inhibition of NFκB significantly increased TNFα-induced cell death in LM7wt and SaOS2wt cells indicating that NFκB has a protective role in LM7 and SaOS2 cells following TNFα treatment. Interestingly, inhibition of NFκB in FADD knockdown cells increased TNFα-induced cell death beyond that observed in NFκB-inhibited wildtype cells (Figures 6B-C). These observations suggest that FADD affects NFκB activation and signaling. Indeed, FADD has previously been reported to affect NFκB activation. For example, FADD has been linked to NFκB activation in Jurkat cells 23, 24 and over expression of FADD in human 293 cells induce NFκB activation 25. Negative regulation of NFκB activation by FADD has also been reported in TNFα-treated cardiomyocytes 26 and FADD suppresses lipopolysaccharide- and IL-1β-induced activation of NFκB in endothelial cells 27. Still, these studies did not investigate the effect of FADD knockdown on NFκB signaling following TNFα treatment.

At the time of publication, only one study was found that reported a link between FADD modulation and sensitivity to TNFα. Khwaja and colleagues reported that inhibition of the FADD/caspase signaling pathway sensitizes leukemia cells to TNFα-induced cell death 28. In the present study, caspase inhibition reversed TNFα-induced cell death in FADD knockdown cells. This opposing observation may be attributed to two factors. First, the cell lines used in the present study differ from the cell lines used in the study carried out by Khwaja and colleagues 28. Second, in the present study, FADD protein expression was reduced by lentiviral-mediated knock down of FADD, while a dominant negative FADD lacking a DED was used in the study carried out by Khwaja and colleagues 28.

Inhibition of XIAP increases TNFα-induced cell death in melanoma 29 and OS 30. This observation is also reported in the current study, indicating a role for XIAP in protection against TNFα-induced cell death in the OS cell lines investigated in this study. XIAP inhibits apoptosis by binding to and inhibiting activated caspases-3, -7 and -9 14. Therefore, following the observation of activated caspase-3 in both LM7wt and LM7fkd cells with significant cell death restricted to LM7fkd cells, we suspected that the NFκB target gene, XIAP, may be involved. It is plausible that although caspase-3 was activated in LM7wt cells, XIAP inhibited caspase-3 from facilitating apoptotic-induced cell death. This would suggest that FADD knockdown inhibits XIAP activity, thus allowing TNFα-induced apoptosis in LM7fkd cells. To investigate the role of XIAP in TNFα-induced cell death in FADD knockdown OS cells, wildtype and FADD knockdown OS cells were pretreated with the XIAP inhibitor, SM-164, followed by treatment with TNFα. While inhibition of XIAP induced TNFα-induced cell death in all wildtype OS cells investigated, the amount of cell death was greater in XIAP-inhibited FADD knockdown OS cells following TNFα treatment (Figure 7A-C). This observation further supports a pro-survival role for FADD following TNFα treatment in OS.

The observation that FADD knockdown increased TNFα-induced cell death in both NFκB-inhibited and XIAP-inhibited cells suggest that FADD has a role in linking TNFα signaling to NFκB-mediated pro-survival pathways. It is plausible that in certain contexts or cancer cells, FADD may serve as an adaptor protein that links TNFα signaling to the pro-survival NFκB pathway. Therefore, when FADD is inhibited by mutation or there is decreased expression of FADD, the effect of TNFα signaling is cell death. In the absence of FADD, the c-Jun N-terminal kinase (JNK) pathway has been suggested as a pathway for TNFα-induced apoptosis 31. Therefore, although not investigated in the current study, it is plausible that the cell death observed in FADD knockdown cells may occur through the JNK pathway.

Cellular status of FADD has been proposed to have clinical relevance as a prognostic indicator for certain cancers. For example, increased phosphorylation of FADD has been implicated in poor prognosis in lung adenocarcinoma 32. Additional observations supporting the pro-survival role of phosphorylated FADD in cancer is the report that inhibition of FADD phosphorylation increases cisplatin-induced cell death in A549 lung cancer cells 33 and the report of increased FADD phosphorylation in T cell lymphomas 34. Furthermore, FADD gene amplification and FADD overexpression has been reported in oral squamous cell carcinoma 35.

Although non-apoptotic functions of FADD were first reported more than 15 years ago, reports of non-apoptotic functions of FADD in cancer is limited and there are no reports on the non-apoptotic functions of FADD in OS. The observation in the present study that FADD knockdown increases TNFα-induced cell death coupled with the observation that inhibition of NFκB in LM7wt cells increases TNFα-induced cell death to similar levels observed in LM7fkd cells supports a regulatory role for FADD in NFκB pro-survival signaling in OS.

## Conclusions

In conclusion, the results of this study reveal a pro-survival function for FADD in OS following TNFα treatment that involves NFκB activation. In addition, the results suggest that FADD is involved in XIAP-mediated protection following TNFα treatment. We base this conclusion on the observation that knock down of FADD increased TNFα-induced cell death (Figure 2B). In addition, inhibition of NFκB resulted in TNFα-induced cell death in LM7wt cells that was similar to that observed in LM7fkd cells (Figure 6B). Furthermore, FADD knockdown increased SMAC mimetic-mediated TNFα-induced cell death (Figure 7A-C). To the best of our knowledge, this is the first report of a pro-survival function for FADD in OS following TNFα treatment. The report presented here of a pro-survival function for FADD in OS and other studies that report pro-survival functions of FADD in other cancers underscore the need to further investigate the multifunctional role of FADD in cancer initiation, progression and survival.

## Abbreviations

DD: death domain
DED: death-effector-domain
FADD: fas-associated protein with death domain
FasL: fas ligand
IkB: inhibitor of NFκB
IKK: IkB kinase
NFκB: nuclear factor kappa B
OS: osteosarcoma
TNFα: tumor necrosis factor-alpha
TNFR: tumor necrosis factor receptor
TNFSF: tumor necrosis factor super family
TRADD: TNFR-associated death domain
TRAIL: tumor necrosis factor-related apoptosis inducing ligand
XIAP: X-linked inhibitor of apoptosis protein

## References

[1] Tran SJ, Tran R, Malipatil NB (2017). Pediatric Osteosarcoma: An Updated Review. Indian J Med Paediatr Oncol.

[2] Misaghi A, Goldin A, Awad M, Kulidjian A (2018). Osteosarcoma: a comprehensive review. SICOT J.

[3] Farfalli GL, Albergo JI, Lobor PA, Smith DE, Streitenberger PD, Pallotta Rodriguez MG, Aponte-Tinao LA (2015). Osteosarcoma lung metastases. Survival after chemotherapy and surgery. Medicina.

[4] Chinnaiyan AM, O'Rourke K, Tewari M, Dixit VM (1995). FADD, a novel death domain-containing protein, interacts with the death domain of Fas and initiates apoptosis. Cell.

[5] Chaudhary PM, Eby M, Jasmin A, Bookwalter A, Murray J, Hood L (1997). Death receptor 5, a new member of the TNFR family, and DR4 induce FADD-dependent apoptosis and activate the NF-kappaB pathway. Immunity.

[6] Schutze S, Tchikov V, Schneider-Brachert W (2008). Regulation of TNFR1 and CD95 signaling by receptor compartmentalization. Nature Review.

[7] Newton K, Harris AW, Bath ML, Smith KG, Strasser A (1998). A dominant interfering mutant of FADD/MORT1 enhances deletion of autoreactive thymocytes and inhibits proliferation of mature T lymphocytes. EMBO J.

[8] Zhang J, Kabra NH, Cado D, Kang C, Winto A (2001). FADD-deficient T cells exhibit a disaccord in regulation of the cell cycle machinery. J Biol Chem.

[9] Zhang R, Liu Y, Hammache K, He L, Zhu B, Cheng W, Hua ZC (2017). The role of FADD in pancreatic cancer cell proliferation and drug resistance. Oncology Letters.

[10] Wang X, Yang L (2008). Tumor necrosis factor and cancer, buddies or foes?. Acta Pharmacol Sin.

[11] Catz SD, Johnson JL (2001). Transcriptional regulation of bcl-2 by nuclear factor kappa B and its significance in prostate cancer. Oncogene.

[12] Kreuz S, Siegmund D, Scheurich P, Wajant H (2001). NF-kappaB inducers upregulate cFLIP, a cycloheximide-sensitive inhibitor of death receptor signaling. Mol Cell Biol.

[13] Turner DJ, Alaish SM, Zou T, Rao JN, Wang JY, Strauch ED (2007). Bile salts induce resistance to apoptosis through NF-kappaB-mediated XIAP expression. Ann Surg.

[14] Deveraux QL, Takahashi R, Salvesen GS, Reed JC (1997). X-linked IAP is a direct inhibitor of cell-death proteases. Nature.

[15] Philchenkov A, Miura K (2016). The IAP Protein Family, SMAC mimetic and Cancer Treatment. Crit. Rev Oncog.

[16] Jia SF, Worth LL, Kleinerman ES (1999). A nude mouse model of human osteosarcoma lung metastases for evaluating new therapeutic strategies. Clin Exp Metastasis.

[17] Fogh J, Fogh JM, Orfeo T (1977). One hundred and twenty-seven cultured human tumor cell lines producing tumors in nude mice. J Natl Cancer Inst.

[18] Vanlangenakker N, Bertrand MJ, Bogaert P, Vandenabeele P, Berghe TV (2011). TNF-induced necroptosis in L929 cells is tightly regulated by multiple TNFR1 complex I and II members. Cell Death Dis.

[19] Xie Y, Hou W, Song X, Yu Y, Huang J, Sun X, Kang R, Tang D (2016). Ferroptosis: process and function. Cell Death Differ.

[20] Tait SW, Ichim G, Green DR (2014). Die another way - non-apoptotic mechanisms of cell death. J Cell Sci.

[21] Linkermann A, Green DR (2014). Necroptosis. N Engl J Med.

[22] Wajant H, Scheurich P (2011). TNFR1-induced activation of the classical NFκB pathway. FEBS J.

[23] Wajant H, Hass E, Schwenzer R, Muhlenbeck F, Kreuz S, Schubert G, Grell M, Smith C, Scheurich P (2000). Inhibition of death receptor-mediated gene induction by a cycloheximide-sensitive factor occurs at the level of or upstream of Fas-associated death domain protein (FADD). J Biol Chem.

[24] Kreuz S, Siegmund D, Rumpf JJ, Samel D, Leverkus M, Janssen O, Hacker G, Dittrich-Breiholz O, Kracht M, Scheurich P, Wajant H (2004). NFkappaB activation by Fas is mediated through FADD, caspase-8, and RIP and is inhibited by FLIP. J Cell Biol.

[25] Hu WH, Johnson H, Shu HB (2000). Activation of NF-kappaB by FADD, Casper, and caspase-8. J Biol Chem.

[26] Chao W, Shen Y, Li L, Zhao H, Meiler SE, Cook SA, Rosenzweig A (2005). Fas-associated death-domain protein inhibits TNF-alpha mediated NF-kappaB activation in cardiomyocytes. AM J Physiol Heart Circ Physiol.

[27] Bannerman DD, Tupper JC, Kelly JD, Winn RK, Harlan JM (2002). The Fas-associated death domain protein suppresses activation of NF-kappa B by LPS and IL-1 beta. J Clin Invest.

[28] Khwaja A, Tatton L (1999). Resistance to the cytotoxic effects of tumor necrosis factor alpha can be overcome by inhibition of a FADD/caspase-dependent signaling pathway. J Biol Chem.

[29] Varfolomeev E, Blankenship JW, Wayson SM, Fedorova AV, Kayagaki N, Garg P, Zobel K, Dynek JN, Elliott LO, Wallweber HJ, Flygare JA, Fairbrother WJ, Deshayes K, Dixit VM, Vucic D (2007). IAP antagonists induce autoubiquitination of c-IAPs, NF-kappaB activation, and TNFalpha-dependent apoptosis. Cell.

[30] Shekhar TM, Miles MA Gupte A, Taylor S Tascone B, Walkley CR Hawkins CJ (2016). IAP antagonists sensitize murine osteosarcoma cells to killing by TNFα. Oncotarget.

[31] Papa S, Zazzeroni F, Pham CG, Bubici C, Franzoso G (2004). Linking JNK signaling to NF-κB: a key to survival. J of Cell Science.

[32] Chen G, Bhojani MS, Heaford AC, Chang DC, Laxman B, Thomas DG, Griffin LB, Yu J, Coppola JM, Giordano TJ, Lin L, Adams D, Orringer MB, Ross BD, Beer DG, Rehemtulla A (2005). Phosphorylated FADD induced NF-kappaB, perturbs cell cycle, and is associated with poor outcome in lung adenocarcinomas. Proc Natl Acad Sci USA.

[33] Schinske KA, Nyati S, Khan AP, Williams TM, Johnson TD, Ross BD, Tomas RP, Rehemtulla A (2011). A novel kinase inhibitor of FADD phosphorylation chemosensitizes through the inhibition of NF-κB. Mol Cancer Ther.

[34] Patel S, Murphy D, Haraiambieva E, Abdulla ZA, Wong KK, Chen H, Gould E, Roncador G, Hatton C, Anderson AP, Banham AH, Pulford K (2014). Increased expression of phosphorylated FADD in anaplastic large cell and other T-cell lymphomas. Biomark Insights.

[35] Chien HT, Cheng SD, Chuang WY, Liao CT, Wang HM, Huang SF (2016). Clinical implications of FADD gene amplification and protein overexpression in Taiwanese oral cavity squamous cell carcinomas. PLOS One.

